# Environmentally Relevant Concentration of Bisphenol S Shows Slight Effects on SIHUMIx

**DOI:** 10.3390/microorganisms8091436

**Published:** 2020-09-19

**Authors:** Stephanie Serena Schäpe, Jannike Lea Krause, Rebecca Katharina Masanetz, Sarah Riesbeck, Robert Starke, Ulrike Rolle-Kampczyk, Christian Eberlein, Hermann-Josef Heipieper, Gunda Herberth, Martin von Bergen, Nico Jehmlich

**Affiliations:** 1 Department of Molecular Systems Biology, Helmholtz-Centre for Environmental Research GmbH – UFZ, 04318 Leipzig, Germany; stephanie.schaepe@ufz.de (S.S.S.); rebecca_masanetz@t-online.de (R.K.M.); sarah.riesbeck@ufz.de (S.R.); ulrike.rolle-kampczyk@ufz.de (U.R.-K.); martin.vonbergen@ufz.de (M.v.B.); 2 Department of Environmental Immunology, Helmholtz-Centre for Environmental Research GmbH – UFZ, 04318 Leipzig, Germany; jannike-lea.krause@ufz.de (J.L.K.); gunda.herberth@ufz.de (G.H.); 3 Laboratory of Environmental Microbiology, Institute of Microbiology of the Czech Academy of Sciences, 14220 Prague, Czech Republic; robert.starke@biomed.cas.cz; 4 Department of Environmental Biotechnology, Helmholtz-Centre for Environmental Research GmbH – UFZ, 04318 Leipzig, Germany; christian.eberlein@ufz.de (C.E.); hermann.heipieper@ufz.de (H.-J.H.); 5 Institute of Biochemistry, Faculty of Biosciences, Pharmacy and Psychology, University of Leipzig, 04103 Leipzig, Germany

**Keywords:** in vitro model, bisphenol S, metaproteomics, short-chain fatty acids, fatty acid methyl ester, intestinal microbiota

## Abstract

Bisphenol S (BPS) is an industrial chemical used in the process of polymerization of polycarbonate plastics and epoxy resins and thus can be found in various plastic products and thermal papers. The microbiota disrupting effect of BPS on the community structure of the microbiome has already been reported, but little is known on how BPS affects bacterial activity and function. To analyze these effects, we cultivated the simplified human intestinal microbiota (SIHUMIx) in bioreactors at a concentration of 45 µM BPS. By determining biomass, growth of SIHUMIx was followed but no differences during BPS exposure were observed. To validate if the membrane composition was affected, fatty acid methyl esters (FAMEs) profiles were compared. Changes in the individual membrane fatty acid composition could not been described; however, the saturation level of the membranes slightly increased during BPS exposure. By applying targeted metabolomics to quantify short-chain fatty acids (SCFA), it was shown that the activity of SIHUMIx was unaffected. Metaproteomics revealed temporal effect on the community structure and function, showing that BPS has minor effects on the structure or functionality of SIHUMIx.

## 1. Introduction

Bisphenols are an initial material in the production of polycarbonate plastics and epoxies resins, and thus can be found in a variety of everyday products, e.g., plastic bottles and boxes used for liquid and food storage or the inner coating of food cans [1,2]. However, a growing number of studies indicate that bisphenols show health-threatening effects on humans [3].

The most commonly applied bisphenol A (BPA) has been classified as endocrine disruptor and has been associated with the development of diseases e.g., diabetes [4]. Due to its endocrine disrupting properties BPA has been banned from products in food packaging and consumer products used by small children and has been added to the EU candidate list for substances of very high concern (SVHC) [5]. BPA is increasingly replaced by structure analogues, including bisphenol S (BPS). However, BPS was also found to impact human health. It was reported that BPS impairs blood functions by affecting blood cells, glucose and cholesterol metabolism and inducing cardiovascular risks in rats [6]. It has also similar estrogenic activity when compared with BPA, although it showed lower acute toxicity in vivo [3]. In 2017, BPS has been added to the Chemicals of High Concern to Children (CHCC) Reporting List in Washington state [7].

Bisphenols are detected up to concentrations of 1000 µg/L in environmental samples such as surface waters of rivers and lakes [8]. They enter the human body through different exposure routes and also find their way towards human gut microbiota. Studies reported the accumulation of BPA and its analogues in the human body [9] and quantified metabolites in blood and urine [10,11,12]. The host–microbiota-interactions in the human gut are essential for human health [13]. This interaction can be effected by environmental chemicals if they support or suppress bacterial growth or if taxonomic composition and functions are effected, both has been shown for bacterial communities in the past [14,15]. Furthermore bacterial growth can also be supported by environmental chemicals due to the provision of its energy or carbon supplying properties mostly due to hydrolytic and reductive reactions [16]. The transformation potential has been proved to mediate BPA and BPS degradation by bacteria in industrial wastewater treatment plants, water, and seawater [17,18].

It was reported that bisphenols can also be toxic to bacteria by destabilizing cell membranes, thereby disturbing its integrity and effecting specifically the membrane permeabilization [19]. Recently, it was observed that bisphenols (especially BPA, BPS, and BPF) are likely to accumulate at bacterial membranes due to their lipophilicity and therefore may lead to disturbances in the cell functioning and to cell destruction [20]. Importantly, bisphenols were also shown to modify microbial composition. In mice, at a concentration of 120 µg/mL BPA solved in DMSO, the alpha- and beta-diversity of the intestinal microbiota was altered by favoring the growth of *Proteobacteria* [21]. In zebrafish, a different concentration of BPA and its analogues including BPS were shown to affect zebrafish gut microbiota [22]. Catron et al. found that different bisphenols alter the intestinal microbiota by changing specific family abundances (e.g., *Neisseriaceae*, *Cryomorphaceae*), with BPS being the least toxic BPA analogues for host development and estrogenicity but the most potent in microbial disruption at a concentration of 45 µM (11.2 µg/mL) BPS solved in DMSO. However, little is known about how the intestinal microbiota is altered on a functional level when exposed to BPS. To our knowledge, concentrations of bisphenols in the human gut have not been measured yet, hence we used the concentration of 45 µM that was previously shown to affect zebrafish gut microbiota to investigate how BPS affected microbial functions.

Recently, we established an in vitro bioreactor model for continuous cultivation of the extended simplified human intestinal microbiota (SIHUMIx) [23]. SIHUMIx comprises of eight species representing a majority of metabolic activities typically found in the human intestine. The cultivation in vitro is highly reproducible and reaches a constant state giving a starting point for stress exposure studies [24]. In this study, we investigated how the exposure of 45 µM BPS in DMSO modulates (i) overall growth, (ii) membrane fatty acid composition, (iii) taxonomic composition, and (iv) functional changes of SIHUMIx.

## 2. Materials and Methods

### 2.1. Simplified Human Intestinal Microbiota—SIHUMIx

The extended simplified human intestinal microbiota (SIHUMIx) consists out of the following eight species: *Anaerostipes caccae* (DSMZ 14662), *Bacteroides thetaiotaomicron* (DSMZ 2079), *Bifidobacterium longum* (NCC 2705), *Blautia producta* (DSMZ 2950), *Clostridium butyricum* (DSMZ 10702), *Clostridium ramosum* (DSMZ 1402), *Escherichia coli* K-12 *(MG1655),* and *Lactobacillus plantarum* (DSMZ 20174) [25]. The cultivation protocol, the growth conditions and the medium ingredients were used as reported (Supplementary material Table S1).

### 2.2. Experimental Set-Up

For inoculation of the bioreactor system, the single strain bacteria were thawed from a fresh glycerol stock two weeks before the experiment started and grown in Brain–Heart-Infusion (BHI) as described (Supplementary material Table S1). Bacteria from three-day old cultures were counted at a Multisizer 3 (Beckman Coulter, Brea, CA, USA) prior to inoculation. 1 × 10^9^ bacteria per strain (a total of 8 × 10^9^ bacteria per 250 mL) were inoculated into the bioreactors (d0). The continuous cultivation was started after 24 h.

**Figure 1:**
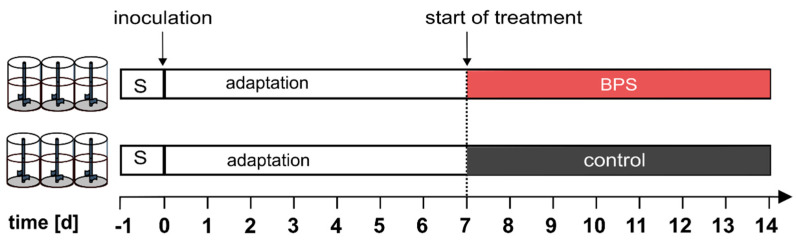
Experimental set-up of the bioreactor run: six bioreactors were inoculated with 1 × 10^9^ cells/250 mL and run for 7 days under continuous culture conditions. On day 7, three bioreactors were exposed to bisphenol S (BPS) resulting in a constant BPS exposure of 45 µM until day 14.

The bioreactor run consists of two phases: (i) The adaption phase where the community established and reached a constant community state (d1–d7) and the treatment phase (d8–d14) in which the effect of 7 days BPS exposure was investigated (Figure 1).

A concentration of 45 µM BPS (Sigma Aldrich, St. Louis, MI, USA) dissolved in DMSO (final concentration in the bioreactor 1%) was applied, since this concentration has been reported to alter the intestinal microbiota taxonomy in zebrafish [22]. It represents a concentration equal to the 4x ADI of BpA in humans. In the control bioreactors, an equal amount of DMSO was added as solvent control. During the whole experiment, samples were taken every 24 h starting the day after inoculation (d1). Community adaption was followed by targeted metabolomics of short-chain fatty acid [26,27]. Microbiota growth was evaluated with determination of absolute biomass. The community structure and function of SIHUMIx was analyzed with metaproteomics on day 5, 6, 7, 8, 12, 13, and 14. Bacteria suspensions were centrifuged at 3200× *g* for 10 min at 4 °C and immediately frozen at −80 °C for subsequent sample analysis.

### 2.3. Microbial Growth

For microbial biomass determination, 4 mL of bioreactor liquid including bacterial cells were centrifuged (5000× *g*, 10 min), washed twice (Phosphate Saline Buffer; PBS), dried in a centrifugal vacuum concentrator (350× *g*, 40 °C; N-Biotek, Bucheon, South Korea) and weighed using a standard precision scale.

### 2.4. Metaproteomics

#### 2.4.1. Protein Extraction

Two mL bioreactor liquid was centrifuged (3200× *g*, 10 min, 4 °C) and the pellet was solved in 1 mL lysis buffer (8M Urea, 2M Thiourea, 1 mM Phenylmethylsulfonylfluorid). Bacteria were disrupted by bead beating (FastPrep-24, MP Biomedicals, Sanra Ana, CA, USA; 5.5 ms, 1 min, 3 cycles) followed by ultra-sonication (UP50H, Hielscher, Teltow, Germany; cycle 0.5, amplitude 60%) and centrifugation (10,000× *g*, 10 min) [28]. The supernatant was used for protein concentration determination using the Pierce^TM^ 660 nm Protein Assay (Thermo Scientific, Thermo Fischer Scientific, Waltham, MA, USA). Ten micrograms of protein lysate was incubated with 25 mM 1,4-dithiothreitol (in 20 mM ammonium bicarbonate) for 1 h and 100 mM iodoacetamide (in 20 mM ammonium bicarbonate) for 30 min. Protein cleaning, cleavage and peptide cleaning were done with hydrophobic Sera-Mag SpeedBead Carboxylate-Modified Magnetic Particles (GE Healthcare, Chicago, IL, USA) as described elsewhere [29]. Proteins were digested with Trypsin (1:50), peptides were eluted with 2% dimethylsulfoxide solved in water without fragmentation. Peptides were solved in 0.1% formic acid for mass spectrometric measurement.

#### 2.4.2. Nano LC MS/MS Measurement

Five micrograms peptides were injected into nano high-performance liquid chromatograph (HPLC) (UltiMate 3000 RSLCnano, Dionex, Thermo Fisher Scientific). Peptide separation was performed on a C18-reverse-phase trapping column (C18 PepMap100, 300 µm × 5 mm, particle size 5 µm, nano viper, Thermo Fischer Scientific), followed by a C18-reverse-phase analytical column (Acclaim PepMap^®^ 100, 75 µm × 25 cm, particle size 3 µm, nanoViper, Thermo Fischer Scientific). Mass spectrometric analysis of peptides was performed on a Q Exactive HF mass spectrometer (Thermo Fisher Scientific) coupled with a TriVersa NanoMate (Advion, Ltd., Harlow, UK) source in LC chip coupling mode. LC gradient, ionization mode and mass spectrometry mode have been used as described before [30].

#### 2.4.3. Data Analysis

Raw data were processed with Proteome Discoverer (v2.2, Thermo Fischer Scientific). Search settings for the Sequest HT search engine were set to trypsin (Full), max. missed cleavage: 2, precursor mass tolerance: 10 ppm, fragment mass tolerance: 0.02 Da. The protein-coding sequences of the eight SIHUMIx strains were downloaded from UniProt (http://www.uniprot.org/), combined and used as database resulting in 29,558 protein sequences. The false discovery rates (FDR) were determined with the node Percolator [31] embedded in Proteome Discoverer and we set to the FDR threshold at a peptide level of <1%. The same threshold was set for the protein FDR (<1%). Protein grouping was performed as described [24]. *GhostKOALA* was used to assign KO numbers from Kyoto Encyclopedia of Genes and Genomes (KEGG) to identified functions of identified protein sequences [32,33]. Protein report from Proteome Discoverer with assigned taxa and functional information from KEEG are provided (Supplementary Material Table S2). Only pathways with sufficient coverage (>10%) on total per sample were used for analysis. For specific pathway abundances only pathways with sufficient relative abundance (>0.1%) and pathway coverage (>3 proteins) per sample were evaluated. Visualization and statistical analysis were done with GraphPad Prism (v8.0.2) using unpaired multiple t-tests per p-value adjustment and are given for taxa and pathways (Supplementary Material Table S2). Principal component analysis was performed using the prcomp function with default setting in R and visualized with ggplot. Statistical protein analysis was performed with MSqRob [34,35]. Protein report from Proteome Discoverer was used as input matrix and pre-processing was applied by log2 transformation, linear regression normalization (Rlr) and no further filtering. Quantification was done by setting treatment per day as fixed settings and bioreactor as random effects, Analysis type: standard, Minimum Fold Change: 0, Number of contrast 1, Contrasts: -1/4 for DMSO control day 8, 12, 13, and 14, respectively and 1/4 for BPS day 8, 12, 13, and 14 respectively. Result table is shown (Supplementary Material Table S3). Volcano plot and boxplots of relevant proteins are provided (Supplementary Material Figure S2).

### 2.5. Short-Chain Fatty Acid Analysis

#### 2.5.1. Metabolite Extraction

For the short-chain fatty acids (SCFAs) analysis the method of Han et al., was modified [26,27]. Briefly, the samples were mixed with acetonitrile to a final concentration of 50% acetonitrile. SCFAs were derivatized with 0.5 volumes of 200 mM 3-nitrophenylhydrazine and 0.5 volumes of 120 mM N-(3-dimethylaminopropyl)-N′-ethylcarbodiimide hydrochloride in pyridine for 30 min at 40 °C. The derivatized SCFA solutions were then diluted 1:50 in 10% acetonitrile.

#### 2.5.2. Measurement and Data Analysis

For identification and quantitation, 50 µL of the diluted SCFA derivatives was injected into the LC-MS/MS system. Chromatographic separation of SCFAs was performed on an Acquity UPLC BEH C18 column (1.7 µm; Waters, Eschborn, Germany) using H_2_O (0.01% formic acid, FA) and acetonitrile (0.01% FA) as the mobile phases. The column flow rate was set to 0.35 mL/min, the column temperature at 40 °C. The gradient elution was performed as follows: 2 min at 15% B, 15–50% B in 15 min, then held at 100% B for 1 min. Finally, the column was equilibrated for 3 min at 15% B. Mass spectrometric analysis of metabolites was performed QTRAP^®^ 5500 (AB Sciex, Framingham, MA, USA). For identification and quantitation, a scheduled MRM method was used, with specific transitions for every SCFA. Peak areas were determined in Analyst^®^ Software (v1.6.2, AB Sciex) and areas for single SCFAs were exported. Normalization and statistics were performed with in-house written *R* scripts.

### 2.6. Lipid Analysis

#### 2.6.1. Lipid Extraction and Derivatization to Fatty Acid Methyl Esters (FAME)

Extraction and derivatization of membrane lipids was carried out according to Bligh and Dyer [36]. Two microliters of bioreactor liquid was taken, centrifuged (3200× *g*, 10 min, 4 °C) and the lipids were extracted with chloroform/methanol/water as described [36]. Fatty acid methyl esters (FAME) were prepared by incubation for 15 min at 80 °C in boron trifluoride/methanol, applying the method of Morrison and Smith, and FAME samples were extracted with hexane [37].

#### 2.6.2. Analysis of Fatty Acid Composition by GC-FID

Analysis of FAME in hexane was performed using a quadruple GC System (HP5890, Hewlett & Packard, Palo Alto, Santa Clara, CA, USA) equipped with a split/splitless injector. A CPSil 88 capillary column (Chrompack, Middelburg, The Netherlands; length, 50 m; inner diameter, 0.25 mm; 0.25 lm film) was used for the separation of the FAME. GC conditions were: injector temperature was held at 240 °C, detector temperature was held at 270 °C. The injection was splitless, carrier gas was helium at a flow of 2 mL/min. The temperature program was: 40 °C, 2 min isothermal; 8 °C/min to 220 °C; 15 min isothermal at 220 °C. The pressure program was: 27.7 psi (=186.15 kPa), 2 min isobaric; 0.82 psi/min (5.65 kPa/min) to the final pressure 45.7 psi; 15.55 min isobaric at 45.7 psi (310.26 kPa). The relative amount of FAMEs were calculated based on peak areas of the total ion chromatograms (TIC). Fatty acids were identified by GC and co-injection of authentic reference compounds obtained from Supelco (Bellefonte, PA, USA).

#### 2.6.3. Data Analysis

The degree of saturation of membrane fatty acids was calculated as described [38] and is defined as the ratio between the two saturated fatty acids (16:0 and 18:0), the two unsaturated fatty acids (16:1cis, 18:1cis 18:1cis). Furthermore, two cyclopropane fatty acids (17*cyc* and 19*cyc*) and the two-branched fatty acids (15:0iso, 15:0anteiso) were detected. The degree saturation of the membrane of SIHUMIx was calculated based on the ratio of saturated to unsaturated fatty acids and cyclopropyl fatty acids (1) and based on the ratio of saturated anteiso- and iso-methyl-branched fatty acids (2) [38,39].

(1) $$\;\mathrm{sat}/\mathrm{unsat}\;+\;\mathrm{cyclo}\;=\;\frac{\left(16:0\;+\;18:0\right)}{\left(16:1\;\mathrm{cis}\;+\;17\;\mathrm{cyc}\;+\;18:1\;\mathrm{cis}\;+\;19\;\mathrm{cyc}\right)},$$

(2) $$\;\mathrm{anteiso}/\mathrm{iso}\;=\;\frac{\left(15:0\;\mathrm{anteiso}\right)}{\left(15:0\;\mathrm{iso}\;\right)},$$

## 3. Results

### 3.1. BPS Does Not Affect Total Biomass

**Figure 2:**
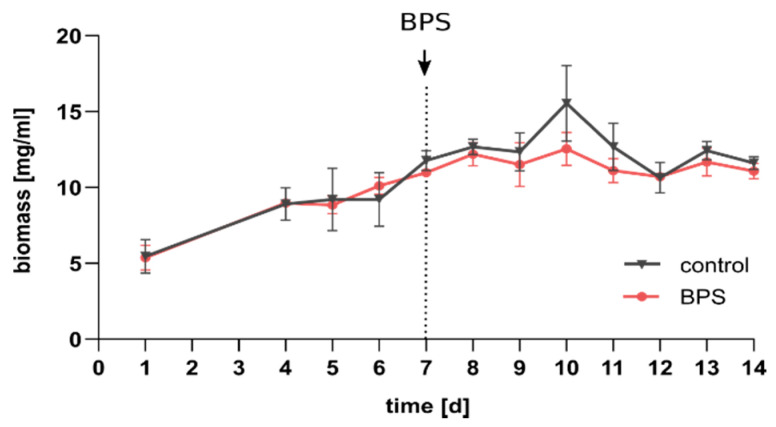
Bacterial growth of simplified human intestinal microbiota (SIHUMIx) indicated by biomass development (mean of *n* = 3 bioreactors).

First, biomass production of SIHUMIx was followed, to investigate growth suppressing effect of BPS exposure. Bioreactors (*n* = 6) were inoculated with 1 × 10^9^ cells/250 mL per species (*n* = 8) and cultivated for seven days until reaching a structural and functional constant state [23]. After sampling on day 7, BPS solved in DMSO or DMSO (equal volume in the control) was spiked into the bioreactors and added to the feed medium to maintain a constant BPS concentration of 45 µM. The redox potential and pH were followed during the experiment (Supplementary Material Figure S1). The bacterial biomass increased starting from day 1 until day 7 to 11.4 ± 0.63 mg/mL in all bioreactors (Figure 2).

After BPS addition, the biomass was slightly lower in the BPS vessels; however, no statistical significance was observed. Except on day 10, the biomass increased and the variation among the three bioreactors vessels was larger compared to the other days. On the last sampling day, the BPS and control bioreactor vessels had similar biomass of 11.6 ± 0.42 mg/mL and 11.1 ± 0.5 mg/mL, respectively.

### 3.2. BPS Does Not Affect SCFA Concentrations

**Figure 3:**
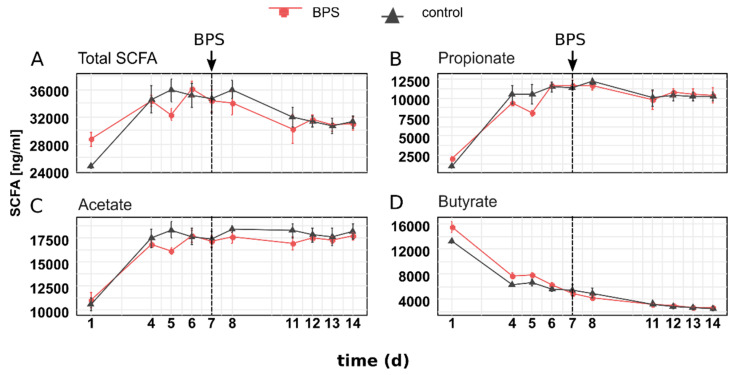
Total (**A**) and individual (**B**–**D**) S simplified human intestinal microbiota (SIHUMIx) concentrations of BPS and control bioreactors (*n* = 3).

To investigate microbial activity, SCFA concentrations were measured. The total amount of all analyzed SCFA and the three highest SCFA propionate, acetate and butyrate, are shown (Figure 3). Less abundant SCFAs are given in the supplement (Supplementary Material Figure S3).

During the establishment of SIHUMIx, the SCFA concentrations were statistically similar in all six bioreactor vessels until day 7. After reaching a comparable concentration at day 7, the total SCFA further deceased after day 7 in all 6 bioreactors, before again reaching a comparable concentration of 31,264.9 ± 1522.8 ng/mL (DMSO control) and 31,030.2 ± 1722.1 ng/mL (BPS), respectively, at day 14. After BPS addition, no statistical significant change was observed on day 8, 11, 12, 13 or 14. Maximal concentration in BPS treated bioreactors of total SCFA (34,314 ± 27,887 ng/mL), acetate (172,467 ± 967 ng/mL), propionate (11,760 ± 1193 ng/mL), isobutyrate (167 ± 8 ng/mL) and isovalerate (168 ± 5 ng/mL) were reached at day 7 and remained unchanged until day 14. 2-methylbutyrate and valerate reached a concentration of 47 ± 4 ng/mL (2-methylbutyrate) and 42.1 ± 10 ng/mL (valerate) at day 7 but decreased afterward towards 24 ± 17 ng/mL and 16 ± 3 ng/mL, respectively at day 14. Butyrate and caproate decreased until day 7 to 4880 ± 642 ng/mL (butyrate) and 1 ± 0.3 ng/mL (caproate), whereas butyrate further decreased to 2613 ± 140 ng/mL at day 14 and caproate concentrations remained unchanged. Overall, no significant difference was found between the BPS treated bioreactor vessels and the control.

### 3.3. BPS Slightly Increases Membrane Saturation Level

**Figure 4:**
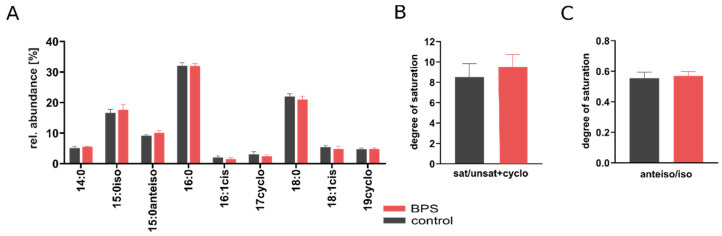
(**A**) The relative abundance of membrane fatty acids in BPS treated and control bioreactors based on the average concentrations at day 13 and 14 (*n* = 6). Degree of saturation of the membrane of SIHUMIx based on the ratio of saturated to unsaturated and cyclopropyl fatty acids (**B**) and on the ratio of anteiso- and iso-methyl-branched fatty acids (**C**). No statistical differences were found between BPS-treated and control samples.

A recent study of Hąc-Wydro et al., reported that bisphenols are likely to interfere with bacterial membranes [20].To validate changes in the lipid composition, the relative abundance of saturated (16:0, 18:0), unsaturated (16:1*cis*, 18:1*cis*), saturated branched-chain fatty acids (15:0*iso*, 15:0*anteiso*) and cyclopropane fatty acids (17:0cyc, 19:0cyc) during the last two days of the experiment were determined (Figure 4A).

The proportion of fatty acids was ranked as followed: C16:0 > C18:0 > 15:0*iso* > 15:0*antiso* > C14 > C18*cis* > C19cyc > C17cyc > C16:1*cis* while no significant difference between BPS exposure SIHUMIx and the control was observed. To investigate the bacterial membrane response to environmental stress, the degree of saturation was calculated as described before [38]. The degree of saturation of sat/unsat+cyclo was moderately but not significantly higher in the BPS exposed SIHUMIx (3.9 ± 0.4) compared to the control (3.6 ± 0.5; Figure 4B). However, no statistical difference was observed in the degree of saturation of *anteiso/iso* branched-chain fatty acids in the BPS treated bioreactors (0.55 ± 0.03) and the control (0.57 ± 0.03; Figure 4C). The sample size necessary to detect significant differences between the BPS-treatment and control was calculated with the following parameter: mu1 = 3.9, mu2 = 3.6, sigma = 0.5, α = 0.05, desired power = 90 (https://www.stat.ubc.ca/~rollin/stats/ssize/n2.html) and showed that 59 replicates are needed.

### 3.4. Metaproteomics Revealed Temporal Effects on the Community, Structure and Functionality

**Figure 5:**
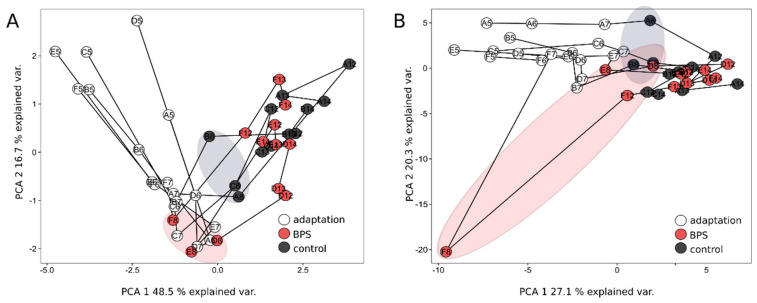
PCA analysis based on the relative protein abundance per species (**A**) and pathway (**B**). Lines connect different time points (day 5, 6, 7, 8, 12, 13, 14) per bioreactor (A, B, C, D, E, F). Ellipses mark the first day after the exposure to BPS.

To assess the species distribution of SIHUMIx, the label-free quantification (LFQ) of species-specific proteins was performed [40]. The list of protein identifications per sample is provided (Supplementary Material Table S2). In all six bioreactor vessels, the relative species abundances were similar on day 7. Between day 8 and day 14, the species abundances slightly changed to day 7 in both, the BPS treated and the control bioreactors, respectively (Supplementary Material Figure S4). Figure 5A shows the Principal Component Analysis (PCA) based on the relative protein intensity per taxa. During the adaptation, all bioreactors developed into the same direction until day 7. After the addition of BPS-DMSO (red ellipse) or DMSO (black ellipse), the bacterial community structure differed on day 8 between the groups as highlighted (Figure 5A). However, after 14 days both community structures developed into a similar structure again (Figure 5A). This was mainly based on the abundance changes of the low abundant (>1%) of SIHUMIx that were more abundant (*E. ramosum*) or less abundant (*B. longum*) in the BPS treated bioreactors on day 8 (Supplementary Material Table S2). After day 8, the species reached similar abundances again, indicating no long-term effect of BPS on the community structure (Figure 5A). As also the control bioreactors differed from day 7 after the DMSO solvent control was added but reached a similar state again at day 14, this difference could be the result of the solvent itself on the bacterial community.

To describe functional changes during BPS exposure, first individual protein abundances were analyzed. In the dataset (*n* = 42 samples), a total of 4,931 different proteins were quantified and fold change between BPS treated and DMSO-control bioreactors were calculated (see Volcano plot in Supplementary Material Figure S2). Sixteen proteins showed significantly higher abundances within the DMSO control bioreactors (*n* = 12, day 8, 12, 13, 14, bioreactor A, B, C) and 30 proteins were significantly higher abundant in the BPS treated bioreactors (*n* = 12, day 8, 12, 13, 14 bioreactor D, E, F). An overview of significantly changed proteins is provided (Supplementary Material Table S3). However, from 46 differently abundant proteins, only 17 were functionally annotated by KEGG. Functions assigned to the proteins higher in the BPS treated bioreactors included a subunit of an Acetyl-CoA carboxylase and a phosphoglucosamine mutase from *A. caccae* (Uniprot accession B0MI45 and B0MGA4), involved in fatty acid synthesis as well as peptidoglycan and lipopolysaccharide (LPS) synthesis, respectively. Also three transporters, two assigned to *B. thetaiotaomicron* (Uniprot accession Q8A992 and Q8A991), one assigned to *B. producta* (Uniprot accession AOA1C7|531) are functional associated with teichoic acid and LPS export. All were less abundant in the DMSO control (Supplementary Material Figure S2).

To investigate weather specific functions of SIHUMIx were affected during BPS exposure, functional categories were determined by metaproteomics. LFQ values of protein groups assigned to a KEGG pathway-level were summed up for each sample. After quality filtering, in the total dataset, 73 different pathways passed quality filtering and were selected for further data interpretation. Principal component analysis (PCA) was performed based on the relative abundance per day (Figure 5B). For biological interpretation, individual pathways were compared (Supplementary Material Table S2). We observed no significant changes in the relative abundances of assigned pathways BPS exposure compared to the DMSO control (Supplementary Material Table S2. Only one bioreactor (F) responded functionally at day 8, which was not the case for the two other biological replicates D and E.

## 4. Discussion

The investigation of bisphenol exposure on different model organisms revealed concerning findings. Effects of BPA and the structural similar BPS were observed in estrogenic activity, serum consumption, and reproduction in rats, mice, or zebrafish [6,41,42]. Recent findings showed a concentration-dependent disruption of the microbial community structure in zebrafish after in vivo exposure to different bisphenol analogues [4,22]. Catron et al., tested different bisphenols at concentrations from 0.2 to 45 µM. Interestingly, they found that microbial disruption was inversely related to host developmental toxicity with BPS being the bisphenol with the highest microbiota-disrupting potential [22]. Considering the fact that the median estimated daily intake of BPS ranges between 0.023–1.67 µg/person [43] and there is clear evidence that gut microbiota are affected by environmental chemicals, potentially affecting health [15], it is of great interest how BPS impacts the human intestinal microbiota.

In this study, we used SCFA analysis and metaproteomics to analyze the impact of BPS on the structure and function of the human intestinal model community. Together with the investigation of membrane fatty acids composition, we obtained deeper insights into how BPS can affect human intestinal bacterial cells. We showed that a 7-day exposure of SIHUMIx to 45 µM BPS had no effect on the community growth or SCFA metabolism compared to the DMSO control. FAME analysis showed that the membrane saturation level was slightly increased after 7 days of BPS exposure compared to the solvent control. This is in agreement with previous findings showing that BPS changes the organization of bacterial membranes [20]. Functional analysis revealed slight differences between the DMSO control and the BPS treated bioreactors, regarding fatty acid, peptidoglycan and LPS synthesis and transport. Furthermore, we observed that SIHUMIx species abundances temporally differed after 24 h of the treatment at day 8 but reached a similar state after 14 days. This temporal response was observed in all bioreactors, indicating an effect of DMSO itself, which might interfered with the function and membranes of SIHUMIx.

### 4.1. Overall Biomass and Activity of SIHUMIx Comparable to DMSO Control

Although most environmental chemicals, such as BPS, are do not target the gut microbiota directly, they can enter the body and might interact with bacteria [14]. As a result, they potentially affect growth or function of bacterial community members.

The biomass development was similar in the control and BPS treated bioreactors, indicating no obvious effect of BPS on the overall growth of SIHUMIx (Figure 2). This is in contrast to BPA, were for single species, it was shown that BPA exposure at high concentrations (>10 mM) resulted in a reduction of total biomass [44], whereas 5 mM showed no effect.

To describe the effect of BPS exposure on the metabolic activity of SIHUMIx, the SCFA production of SIHUMIx was analyzed. Fermentation products such as SCFA play a beneficial role for the host health and changes would directly impact host metabolism [45,46]. In BPA exposed rats (200 µg/kg body weight/day), a decrease in SCFA acids was already shown [47]. So far, this is the first time that the effect of BPS on SCFA acid production of intestinal bacteria outside of a host has been described. Enzymes from carbohydrate metabolism involved in SCFA synthesis were not affected by BPA exposure. Although the concentration of the SCFA of SIHUMIx reached a comparable amount on day 7, it decreased after the treatment in the DMSO control and the BPS treated bioreactors. However, no changes were observed in the BPS treated bioreactors compared to the control (Figure 3A–D). This suggests that the solvent itself affected the fermentation capacity of SIHUMIx.

### 4.2. BPS Exposure Slightly Increased Membrane Saturation

Since Hąc-Wydro et al., revealed that bisphenols are likely to accumulate at bacterial membranes and therefore may lead to destruction alteration in the membranes of SIHUMIx were investigated. Bacteria can alter their cell membrane fluidity by changing the protein content, the phospholipid head groups or the fatty acid composition in the lipid bilayers [19]. The bacterial lipid metabolism, including regulation, structure and biosynthetic machinery of fatty acid synthesis in bacteria, showed extremely high diversity [48]. SIHUMIx is dominated by the Gram-negative bacterial species *B. thetaiotaomicron* and *E. coli* and Gram-positive *B. producta* (Supplementary Material Figure S2A). In *B. thetaiotaomicron* the main fatty acids are branched-chain fatty acids from C13 to C17 whereas saturated C16:0 is present in a low amount [49,50]. In strains closely related to *B. producta* the dominant lipid is C16:0, followed by C18:0 C18:1 *cis* [51,52]. Together the FAME profile of *B. thetaiotaomicron* and *B. producta* correspond to the FAME profile of SIHUMIx, were saturated (C16:0 > C18:0) and branched-chain (C18:0 > C15) fatty acids were the most abundant. However, studies analyzing the membrane composition of single strains can only partly be compared to findings in communities since lipid profiles in terms of quantitative and qualitative distribution differ when bacteria grow in communities [53].

As the change in fatty acid saturation is known as a long term adaptation, fatty acid composition at the last days after the begin of the exposure were compared [54]. Bacterial membranes consist of saturated and unsaturated fatty acids. *Cis*-unsaturated fatty acids can be transformed into cyclopropyl fatty acids [55], leading to the conversion of 16:1 *cis* to 17:0 cyclopropane (17:0cyc) and 18:1 *cis* to 19:0 cyclopropane (19:0cyc). Bacterial membranes also consist of branched-chain fatty acids. Differences in the amount of *iso*-branched chained fatty and *anteiso*-branched-chain fatty acids have been described in bacteria from contaminated environments since they directly affect membrane fluidity [54]. To validate weather BPS caused changes in the membrane composition of SIHUMIx, fatty acids methyl esters (FAME) were measured (Figure 4B).

When comparing individual FAME abundances, membrane composition in terms of saturated to unsaturated and cyclopropane fatty acids have not been clearly changed between the BPS treated bioreactors and the control. In addition, the degree of saturation calculated by *anteiso/iso* ratio of branched fatty acids was not affected. Bacteria are known to increase their *anteiso/iso* ratio, leading to a more rigid membrane and counteracting the fluidity to resist environmental stress [39]. However, when calculating the degree of saturation of the sat/unsat+cyclo, the level was moderately but not significantly increased in the BPS treated bioreactors.

Generally, the degree of saturation increases with the incorporation of saturated fatty acids in the bacterial membrane, resulting in greater membrane stability able to resist the toxic effects of external stressors [56].

The enzymes involved in fatty acid synthesis vary between bacterial species but have been extensively investigated in *E. coli* [48,57,58]. The regulation of the synthesis rate and the product structure is influenced by various enzymes in the process [48]. If BPS exposure increases the synthesis of fatty acids to more saturated and branched-chain fatty acids or if it causes a conversion of the fatty acids already present in the membranes, it needs to be addressed in further studies.

### 4.3. SIHUMIx Showed Treatment-Dependent Temporal Responses

DMSO is often used in biological research to solve hydrophobic compounds [59]. However, DMSO might be able to directly affect bacterial cells itself. It has been reported that it affects the structure and properties of cell membranes, even at low concentrations [60]. We assumed that the observed temporal changes were caused by the DMSO itself rather than the BPS as reported previously [61] due to no significant differences between the BPS treatment and the control. DMSO influences the packing of hydrocarbon chains of lipid as a result of the dehydration of membrane surface [60]. This means that both DMSO and BPS might cause changes in the bacterial membrane saturation, which cannot be distinguished within this experimental set up.

Metaproteomics revealed that species abundances slightly fluctuated during the exposure (days 7 to 14) in both, BPS treated and control bioreactors. When comparing the relative species abundances at day 8, it was shown that the low abundant species *E. ramosum* and *B. longum* were either less or higher abundant in the BPS treated bioreactors, but with no statistical significance (Supplementary Material Table S2). At day 14, the individual species abundances reached again a comparable cell number. This led us to the conclusion that the microbes showed a temporal response to the treatment. SIHUMIx responded to the treatment, but reaches a constant state as reported previously [24]. This may still indicate that microbes are affected, but can cope with chemicals occurring in the gut. Previous findings revealed disruption of the microbial structure during BPS-DMSO exposure [22]. Catron et al., reported that the growth of the family *Cytophagaceae* was affected by BPS exposure in zebrafish. However, these findings can only partly be compared since the zebrafish intestinal microbiota differ compared to the gut microbiota found in humans. As SIHUMIx does not consist of a species belonging to *Cytophagaceae*, it most likely does not include BPS growth-sensitive species.

When comparing KEGG-pathways, BPS showed no specific effect on SIHUMIx on the functional level (Figure 5B). PCA analysis showed that all bioreactors differed at day 8 from day 7 but reached a similar state again at day 14. Changes in the proteins belonging to three SIHUMIx species (*A. caccae*, *B. thetaiotaomicron*, *B. producta*) could be found, when fold change of the individual protein abundances were compared. Three transporters and two proteins involved in fatty acid, peptidoglycan and LPS export and synthesis were increased in the BPS treated bioreactors, compared to the DMSO control at day 8 (Supplementary Material Figure S3).

The outer layer of the bacterial membrane functions as a barrier to protect the cells from external influences and preventing environmental chemicals from reaching the cytosol. As a typical stress response bacteria modify the outer membrane either by alteration of the membrane structure or by accumulation of hydrophobic substances [62].

Both proteins upregulated in the fatty acid, peptidoglycan and LPS synthesis are assigned to *A. caccae*. Enhanced production of fatty acids could be a coping mechanism for disturbances in the lipid membrane, likely by changing the fluidity of the membrane, as shown in our finding of moderate increase in degree of saturation of sat/unsat+cyclo. Bacteria with reduced LPS are more sensitive to antibiotics and therefore more vulnerable in changing environments [63].

The upregulated ATP binding cassette (ABC) transporter from *B. producta* is the ATP binding region of a two component transport system, involved in the export of wall teichoic acids (WTAs) [64]. Deficiency of WTAs leads to increased temperature sensitivity, can induce defective septum initiation, promoting the generation of round offspring of bacterial cells and influence biofilm forming capacity [65,66,67].

Furthermore, two ABC transporter subunits from the gram negative bacterium *B. thetaiotaomicron* were overrepresented in the BPS treated SIHUMIx community. Both are associated with ABC type-2 transporter units, which are assigned with exporting LPS or teichoic acids from the cytosol for integration into the cell membrane or wall [68]. Based on these findings an increased production and export of LPS, peptidoglycan and WTAs, indicate the need of cell envelope modification during BPS treatment.

The integrity of the bacterial membrane and the homeostasis of various overall cell envelope components are critical for growth and the viability of bacteria. It requires a balance between synthesis of peptidoglycan, phospholipids and LPS [69]. Due to changing the lipid, phospholipid, and glycan metabolism, SIHUMIx might adapted the composition of the membrane to resist organic chemical toxicity. However, functional changes were restricted to single proteins and no functional differences were found when comparing functional pathways of the BPS exposed bioreactors to the DMSO control. As both BPS and DMSO have been reported to affect bacterial membranes, solvent tolerance of the model system should be taken into account in future studies to investigate bisphenols.

## 5. Conclusions

It was shown that the overall growth of SIHUMIx remained unchanged and SCFA production was unaffected by the exposure to 45 µM BPS. Changes in individual membrane fatty acid composition could not be clearly distinguished; however, the adaptation and saturation level of the membranes slightly increased during BPS exposure. Metaproteomics revealed temporal functional and structural response to the treatment, independent of BPS exposure. This is the first study investigating the function of intestinal bacteria after BPS exposure when cultivated in continuous bioreactors. At the tested concentration of 45 µM BPS, we observed changes that were restricted to the bacterial membrane indicating that through their adaptation no key physiological function of intestinal bacteria was affected. Due to the capability of bisphenols to accumulate in the human body it is necessary to test the effects of a range of concentrations. It is still of interest to evaluate the impact of other substitutes used for BPA, taking into account that the structural analogy could imply similar effects. The exposure of chemical mixtures could uncover cooperative effects on the gut microbiota and contribute to a more environmentally relevant picture.

## Supplementary Materials

The following are available online at https://www.mdpi.com/2076-2607/8/9/1436/s1, Supplementary Material Table S1: Growth conditions and media. Supplementary Material Table S2: Metaproteomics. Supplementary Material Table S3: MSqRob protein report. Supplementary Material Figure S1: pH and redox potential of CIM during the bioreactor run (quality control). Supplementary Material Figure S2: Volcano plot. Supplementary Material Figure S3: SCFA concentrations. Supplementary Material Figure S4: Relative protein abundance of SIHUMIx species.
