# Supplementary material for: Environmentally Relevant Concentration of Bisphenol S Shows Slight Effects on SIHUMIx
Source: Microorganisms. 2020 Sep 19;8(9):1436. doi: 10.3390/microorganisms8091436 (PMC7564734; doi:10.3390/microorganisms8091436)
Supplement: Supplementary file 1 [file microorganisms-08-01436-s001.zip › Supplementary_Material Figure_S1_pH_redox_potential.docx]

**Supplementary Material Figure S1: pH and redox potential of CIM during the bioreactor run (quality control)**

Figure S1: pH and redox potential of the Complex Intestinal Media (CIM) during the bioreactor run based in the average (with SD) of the three control and BPS treated bioreactor vessels, respectively.
