# Supplementary material for: Environmentally Relevant Concentration of Bisphenol S Shows Slight Effects on SIHUMIx
Source: Microorganisms. 2020 Sep 19;8(9):1436. doi: 10.3390/microorganisms8091436 (PMC7564734; doi:10.3390/microorganisms8091436)
Supplement: Supplementary file 1 [file microorganisms-08-01436-s001.zip › Supplementary_Material Figure_S2_Volcanoplot.docx]

**Supplementary Material Figure S2: Robust statistical inference for quantitative LC-MS proteomics** (**MSqRob) result plots**


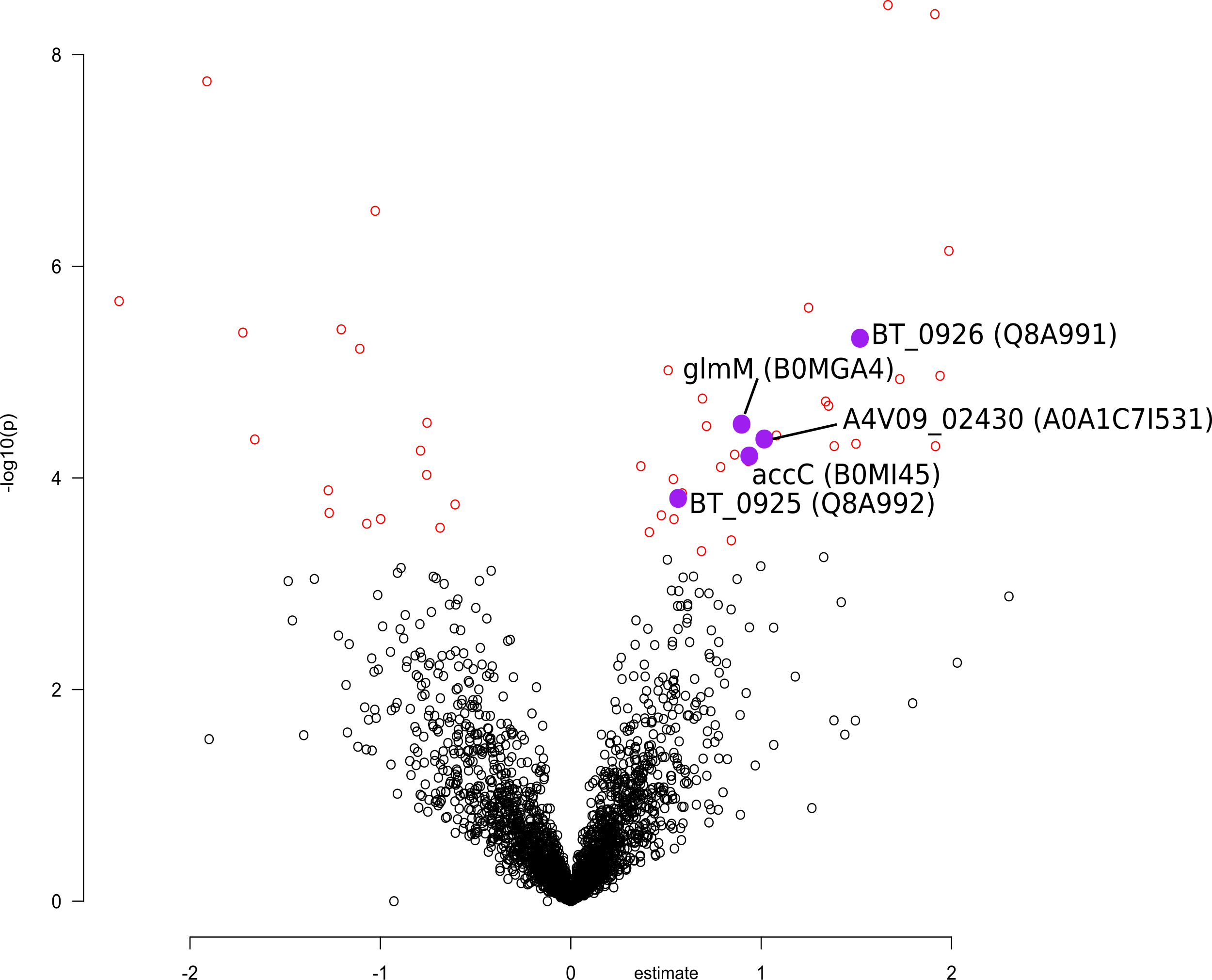


Figure S2: Volcanoplot based on protein abundances. Estimate= Log2(X/Y). Marked are the proteins that are in association with bacterial membrane transports or structure adaptation.
