# Supplementary material for: Environmentally Relevant Concentration of Bisphenol S Shows Slight Effects on SIHUMIx
Source: Microorganisms. 2020 Sep 19;8(9):1436. doi: 10.3390/microorganisms8091436 (PMC7564734; doi:10.3390/microorganisms8091436)
Supplement: Supplementary file 1 [file microorganisms-08-01436-s001.zip › Supplementary_Material Figure_S3_SCFA_concentrations.docx]

**Supplementary Material Figure S4: SCFA concentrations over time**


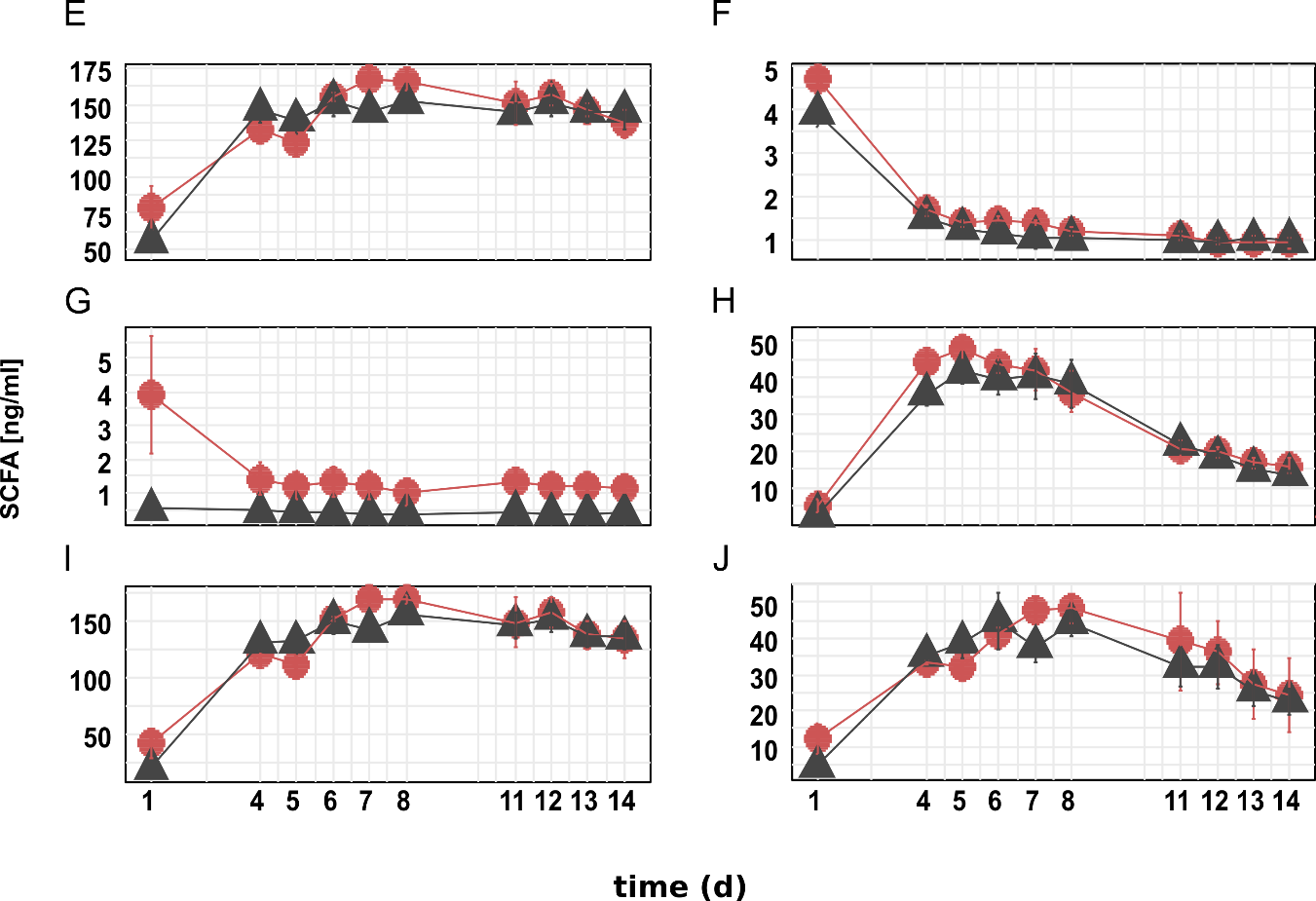


Figure S2: Individual (E-F) SCFA concentrations of BPS and control bioreactors (n=3).
