# Supplementary material for: Environmentally Relevant Concentration of Bisphenol S Shows Slight Effects on SIHUMIx
Source: Microorganisms. 2020 Sep 19;8(9):1436. doi: 10.3390/microorganisms8091436 (PMC7564734; doi:10.3390/microorganisms8091436)
Supplement: Supplementary file 1 [file microorganisms-08-01436-s001.zip › Supplementary_Material Figure_S4_Species.docx]

**Supplementary Material Figure S3: Relative species abundances**

A


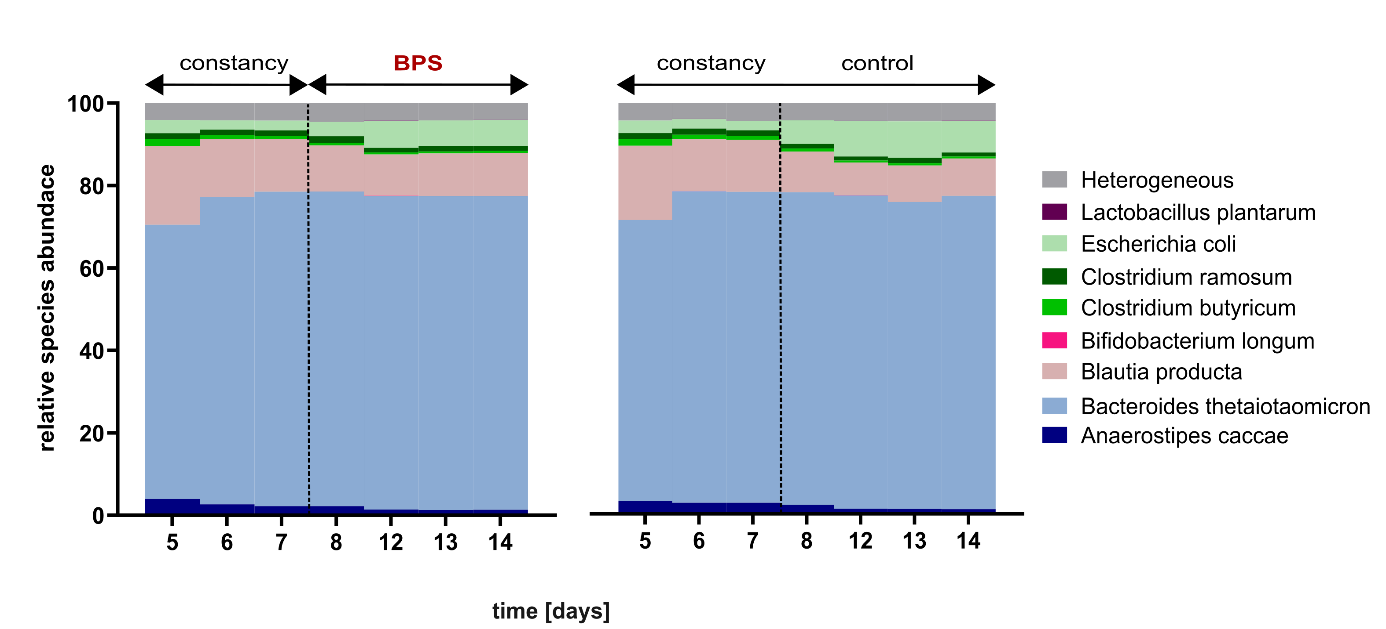


B

C

Figure S2: Relative species abundance as stacked bars (A) and individual species abundances in BPS treated (B) and control (C) bioreactors.
